# The influence of the ownership effect on Chinese children's learning performance: a comparative study between choosing and assigning ownership acquisition styles

**DOI:** 10.3389/fpsyg.2025.1610216

**Published:** 2025-10-09

**Authors:** Taiping Li, Baoquan Lu, Huifen Wu

**Affiliations:** ^1^School of Education, Huazhong University of Science and Technology, Wuhan, Hubei, China; ^2^School of Education and Psychology, Hubei Engineering University, Xiaogan, China

**Keywords:** ownership effect, interdependent self, learning, Chinese children, memory

## Abstract

The cultural context can regulate the ownership effect, thus affecting learning performance and object memory. Previous studies have discussed the influence of ownership effect on children's learning based on the choosing and assigning ownership acquisition style. However, for Chinese children growing up in an interdependent cultural context, it is unclear the differences in the impact of ownership effects on learning performance in the two ownership acquisition styles when intimate others are involved in the learning process. In this study, an independent two-factor mixed experimental design was adopted: 2 (ownership acquisition style: choosing and assigning) × 3 (ownership condition: self, mother/friend/teacher, and others). Each experiment recruited approximately eighty third-grade Chinese students aged 7–9 years old. They were asked to place a series of pictures of themselves, intimate others (mother, friend, or teacher), and strangers in baskets according to color cues (assigning) or self-choice (choosing). They then completed a free-recall task. A repeated-measures analysis of variance was conducted on the correct free recall rate, and the results were as follows: Performance under the chosen ownership acquisition style was better than that under the assigned ownership acquisition style. There was no significant difference between the learning performance in the condition of mother and self-ownership, but both were higher than the learning performance in the condition of others-ownership. Learning performance in the self-ownership and friend-ownership conditions were significantly higher than that in the others-ownership condition, and performance in the self-ownership condition was significantly higher than that in the friends-ownership condition. Performance in the self-ownership condition was significantly higher than that in the teacher-ownership and others-ownership conditions, whereas the performance in the teacher-ownership condition was not significantly different from that in the others-ownership condition. Our findings showed that, for Chinese children, mothers' involvement in children's learning performance was just as beneficial as self-involvement in the ownership teaching situation. Although learning performance in the condition of friend-involvement was not as good as that for self-involvement, it can improve learning performance. However, learning performance was the poorest in the teacher-involvement condition. This research implicated ownership teaching as a teaching strategy that should be widely disseminated in interdependent cultural contexts, especially ownership teaching methods based on choosing. The simplicity, efficiency, and economy of its operation process will inspire educational researchers to fully explore the potentially more valuable tools that may exist in this “educational toolbox.” In addtion, it is necessary to fully leverage the roles of family education and peer-assisted learning in Chinese children's education. This also indicates that in the process of educational reform and policy formulation for Chinese children, the influence of culture should be fully considered.

## 1 Introduction

The ownership effect refers to individuals giving preference to self-owned items and perceiving them as more valuable (Beggan, 1992). Research has shown that the learning and memory performance is significantly better in the self-ownership condition than in others-ownership condition through the creation of ownership situations (Cunningham et al., 2008, 2011, 2013, 2018). In recent years, the ownership effect has received considerable attention in many disciplines, including psychology, management, economics, consumer behavior, and law. This effect suggests that the human valuation of objects is not completely rational (Kahneman, 2003) and may be biased according to the perceiver's attitude and feelings toward the object (Beggan, 1992). Cunningham et al. (2018) were the first to apply the ownership effect to educational activities. Through two experiments, they examined how creating an ownership teaching context during the teaching process could enhance children's learning performance by inducing the ownership effect. In Experiment 1, children aged 7–9 and the experimenter were required to take turns choosing from among nine cards (new graphics composed of meaningless syllables and a line segment) for their shopping carts. After choosing, the children's shopping carts contained three cards (self-ownership condition), the experimenter's shopping cart contained three cards (other-ownership condition), and three cards were not selected and remained on the table (non-ownership condition). The subsequent free recall test indicated that learning performance in the self-ownership condition was significantly higher than in the other-ownership and non-ownership conditions, and learning performance in the other-ownership condition was significantly higher than in the non-ownership condition. Experiment 2 replicated this finding from Experiment 1 by assigning cards to the children rather than allowing them to choose freely and included a control group as the baseline. The results showed that there were no significant differences in learning performance among the three ownership conditions (left, middle, and right ownership) in the control group, whereas there were significant differences in learning performance among the three conditions (self, others, and non-ownership) in the ownership group. Moreover, when comparing the learning performance in the three conditions of the ownership group with the control group, it was found that the learning performance in the self- and others-ownership conditions was significantly higher than that in the control group, while the non-ownership condition showed no significant difference from the control group. This indicates that the self-ownership condition does not reduce the learning performance of the other ownership conditions at the cost of improving children's learning performance. This suggests that the ownership effect can be used to enhance children's learning performance.

The ownership situation created to control the induced ownership effect is similar to a game, which can improve students' participation and attention, thereby enhancing learning performance. Accordingly, researchers add the un-ownership condition. Results of these studies reveal that, when ownership acquisition is either chosen or assigned, learning performance in the self-ownership condition is significantly better than in the others-ownership condition, and both are significantly better than in the un-ownership condition. This indicated that the ownership effect caused by the two ownership acquisition styles is conducive to improving children's learning performance. Improved learning performance in the self-ownership condition did not decrease compared with the others-ownership condition (Cunningham et al., 2018; Wu et al., 2019). Simultaneously, these studies suggest that the effect of the teaching situation can fully mobilize students' self-enthusiasm and promote improvements in learning performance. In addition, Wu et al. (2020) systematically summarized the advantages of the ownership effect when applied to children's learning processes to improve their learning performance. They highlighted the unique nature of learning forms and effects, the applicability to different types of learning materials and children of younger ages, and the efficiency and operability of learning situations.

Previous studies used healthy children as participants, with recognition (Cunningham et al., 2018) and free recall (Wu et al., 2019) as variables. Two independent behavioral experiments (Experiment 1, based on choosing ownership acquisition style, and Experiment 2, based on assigning ownership acquisition style) were conducted to verify whether the ownership effect was beneficial for improving children's learning performance (Cunningham et al., 2018; Wu et al., 2019). In addition, many studies have used children with autism to explore differences in the impact of the ownership effect based on choosing and assigning ownership acquisition styles on children's cognition. The results showed that healthy children exhibited the ownership effect under both choosing-based and assigning-based ownership acquisition styles, while children with autism only exhibited the ownership effect under the choosing-based style (Xu, 2022; Wuyun et al., 2020). Additionally, the ownership effect still occurred even in the fictional self-choosing style (Wuyun et al., 2020). Although previous studies have explored the differences in the ownership effect between healthy children and children with autism under the choosing and assigning ownership acquisition styles, few researchers have adopted a mixed experimental design to compare the differences in the ownership effect under the two different ownership acquisition styles. This has important theoretical and practical significance for the selection of teaching methods in ownership education for healthy children.

Ullmann-Margalit and Morgenbesser (1977) distinguished between picking and choosing to better illustrate the self-fulfilling aspect of choice. According to these researchers, picking does not necessarily express one's desires or preferences, but choice involves an opportunity to meaningfully fulfill one's desires or preferences. This distinction highlights the possibility that, in some experiments, the choices presented to participants allow them to choose but have no effect on their autonomy because choices without preferences do not affect people's interests, will, goals, and values; therefore, some studies have suggested that picking is less motivating than choosing (Rescher, 1960). Some studies found that self-choosing conditions (based on choice) result in higher scores for perceived external agency (Barlas et al., 2018) than mandatory assigned conditions based on assigned conditions (Sebanz and Lackner, 2007; Sidarus et al., 2017). Although evidence suggests that both free- and forced-choice conditions can lead to the expected outcomes of action plans (Gaschler and Nattkemper, 2012; Hommel et al., 2017), some studies have shown that in forced-choice environments, expectations significantly decrease (Herwig and Horstmann, 2011). Making personal choices is a decisive factor of agency and is regarded as the core of self-awareness (Haggard, 2003; Marcel, 2003). Once individuals are given an opportunity to make a choice, even an insignificant choice is sufficient to enhance their memory. Some researchers believe that choice can promote self-focus (Wenzlaff and LePage, 2000), thereby enhancing the memory of the choice outcome (Cloutier and Neil Macrae, 2008; Knoblich and Prinz, 2001; Repp and Knoblich, 2004). Previous studies have shown that choice can influence the type of information recalled regarding choices and ignored options (Dellarosa and Bourne, 1984; Marcel, 2003). Specifically, when options are assigned rather than chosen by the individual, no bias toward “choice-supportive” features is found in the individual (Marcel, 2003). This bias is believed to be driven by an individual's positive expectations and associations with the chosen items. Self-Determination Theory posits that humans tend to interact with the environment in a way that promotes learning and mastery and that autonomy, competence, and belongingness are the three fundamental needs that constitute people's intrinsic motivation (Ryan and Deci, 2000a). Providing choices may be the most obvious way to support an individual's sense of autonomy. A social environment that meets these needs enhances intrinsic motivation (Ryan and Deci, 2000a). Supporting autonomy and intrinsic motivation leads to other adaptive outcomes, including improved performance, learning, and even better health levels. Moreover, Self-Determination Theory holds that choice leads to positive motivational and performance results (Deci and Ryan, 1980, 1985; Ryan and Deci, 2000b). That is, if an activity involves a personal choice or provides the opportunity to make a choice, then people will have stronger intrinsic motivation to persist in a task. Providing choices increases the sense of personal control (Rotter, 1966; Taylor, 1989). Finally, the degree of self-involvement stimulation affects the size of the self-reference effect. Although the ownership effect can be entirely based on attention capture or fine processing of self-concept (Cunningham et al., 2013; Ross et al., 2011), the strongest ownership effect may arise when an individual considers themselves the agent of their own choice, at which point there is higher arousal and participation (Cunningham et al., 2011). Self-involvement (based on the acquisition of ownership through choice) intensifies the ownership effect, meaning that choosing an item for oneself has a particularly strong ownership effect. The strongest ownership effect may be driven by the combination of self-reference and choice. Choice may not a necessary component of ownership, but it is typically related to real-world ownership and exacerbates the ownership effect in the individual (Cunningham et al., 2011). In conclusion, we introduce the ownership effect into the field of children's learning and propose the following research hypothesis, Hypothesis 1: The ownership effect based on the acquisition style of ownership through choice is significantly more effective for children's learning performance than the ownership effect based on the assigned ownership acquisition style.

There are significant differences in the self-concept between individuals from Eastern and Western cultures. Western cultures emphasize an independent self-concept, portraying the self as a unique individual distinct from others, whereas Eastern cultures emphasize an interdependent self-concept, representing the self as interconnected with others, especially close individuals (Sittenthaler et al., 2015; Markus and Kitayama, 1991). This cultural difference is reflected in the memory advantage of self-reference. Previous studies using the trait-word evaluation paradigm found that although Eastern and Western individuals are the same in terms of their memory of words related to the self being significantly better than those related to strangers, Chinese participants showed similar memory preferences for words related to the self and those related to their mothers, while Western participants did not show this difference (Sui et al., 2014). This effect may be influenced by self-knowledge because the trait word evaluation paradigm requires participants to evaluate the relevance of words to themselves and others separately. The Chinese participants were more likely to have knowledge of important others that overlapped with their self-representation. The ownership-based research paradigm avoids the influence of self-knowledge, thereby providing a more objective examination of individuals' self-representations in different cultures. Sparks et al. (2016) adopted the ownership paradigm and found significant mother—and friend-ownership effects (i.e., a relational others-ownership effect) among individuals growing up in Asian cultural contexts. In other words, the learning and memory effects of materials under the self-ownership condition were significantly better than those under the relational other-ownership (mother, friend, etc.) and stranger conditions. Conversely, the learning and memory effects of materials in the relational other-ownership condition were significantly better than those in the strange other-ownership condition. These results also showed no significant difference between the learning and memory effects of materials in the self-ownership and intimate other-ownership conditions; however, both were significantly better than the learning and memory effects of materials in the strange other-ownership condition. On the other hand, for individuals growing up in Western cultures, learning and memory performance in the self-ownership condition was significantly better than in the other-ownership condition (including relational and strange others), showing a significant ownership effect. These results suggest that cultural context can regulate the ownership effect, thereby affecting learning performance and object memory.

Mead, the founder of the symbolic interactionism school, and Cooley, a sociologist, pioneered the exploration of the formation of the self, emphasizing that the self can only be produced and exist through continuous interaction with society (Mead, 1999). There are three kinds of social interactions during childhood: parent—child, peer, and teacher—student interactions. These three social relations have a profound impact on children's self-schemas, and children's learning process involves more than just the teachers. In recent years, educators have increasingly emphasized the importance of parental (Li and Hu, 2021) and peer (Liu and Guo, 2020) involvement in Chinese children's learning processes. In addition, from the perspective of culture and self, the individual's self-concept in the Eastern cultural context has the characteristic of interdependence (Kitayama and Uskul, 2011), which has been confirmed by studying behavior (Yuan, 2007; Zhou and Su, 2008; Wu and Zhou, 2013; Zhao et al., 2014) and through brain imaging (Zhu et al., 2007; Feng et al., 2013). The ownership effect is one type of self-processing bias effect (Sparks et al., 2016). A functional near infrared spectroscopy study found that the left dorsal lateral prefrontal cortex is associated with a learning advantage related to self-processing (Li and Hu, 2021). Research on the self-processing bias of Chinese children found no significant difference between the memory performance of Chinese children in the self- and mother-correlation conditions, both of which were significantly higher than in the friends and teachers condition. Memory performance in the friends and teachers condition was significantly higher than in the others condition. This indicates that Chinese children's selves also have interdependent characteristics and include close relationships with others, such as mothers, friends, and teachers (Yuan, 2007).

This study introduces the concept of the ownership effect in the field of children's learning in China. Using the experimental materials and paradigms from Wu et al. (2019), three experiments were conducted by choosing and assigning ownership acquisition styles: self-ownership, mother/friend/teacher ownership, and unfamiliar ownership. These experiments were designed to explore the impact of the ownership effects triggered by the teaching scenarios of mother/friend/teacher ownership on children's learning performance. Previous studies using adult participants and the self-reference effect research paradigm have shown whether it is the self-reference effect (Zhu et al., 2007; Feng et al., 2013; Zhao et al., 2014) or the ownership paradigm (Sparks et al., 2016). In both cases, the results have indicated that the memory effect in self-related conditions is not significantly different from that in mother-related conditions, and both are significantly higher than that in conditions related to unfamiliar others, demonstrating a clear mother reference effect. This further indicates that the self of Chinese people belongs to an interdependent structure, including close others such as mothers. However, in a study with 4–year-old children using the ownership paradigm, Wu et al. (2021) found that the memory effect in self-ownership conditions was significantly higher than that in mother-ownership conditions. Previous studies have found that for children over 7 years old, the self-processing bias is not affected by the abstractness of learning materials and tasks (Halpin et al., 1984; Pullyblank et al., 1985), indicating that children's self-concept tends to be complete at this age and can show a stable ownership effect (Zhou et al., 2010). Therefore, in this study, 8–9-year-old children were selected as research participants to explore the impact of different ownership acquisition styles on the learning performance of Chinese children when mothers are involved. Hypothesis 2 states that regardless of ownership acquisition style based on choice or assigning, there is no significant difference in learning performance between the self- and mother-ownership conditions, and both are significantly higher than the other-ownership conditions.

Previous studies that used the classic self-reference effect research paradigm found that the memory performance of Chinese adults in the self- and friend-related conditions was significantly higher than that in the other-related condition and that there was no significant difference in memory performance between the self- and friend-related conditions (Guan and Chi, 2006; Wu and Zhou, 2013). However, studies using the incidental encoding research paradigm have found no significant differences in memory performance between self- and friend-related conditions (Wu and Zhou, 2013). The incidental encoding research paradigm refers to the establishment of a connection between the self and the stimulus without requiring the participants to make explicit evaluations of the stimulus features related to the self or without an explicit connection between the self and the stimulus features being encoded. In other words, in tasks in which the self is associated with the stimulus, the stimulus is encoded in an incidental manner (Cloutier and Neil Macrae, 2008; Cunningham et al., 2008). The ownership paradigm is an incidental encoding research paradigm. A study on children found that memory performance in self-related conditions was significantly higher than in friend-related and stranger-related conditions, and memory performance in friend-related conditions was significantly higher than in stranger-related conditions (Yuan, 2007). This study explores the impact of the ownership effect triggered by friends' involvement in different ownership acquisition styles on the learning performance of Chinese children. Hypothesis 3 states that regardless of ownership acquisition style based on choice or assignment, the self-ownership condition significantly outperforms the friend-ownership condition, and the friend-ownership condition significantly outperforms the other-ownership condition.

Wuyun et al. (2020) employed the ownership research paradigm and found that the memory effect on healthy children in the self-ownership condition was significantly better than that in the teacher-ownership and unfamiliar other-ownership conditions. There was no significant difference in the memory effect in the teacher-ownership condition or in the memory effect in the unfamiliar other-ownership condition. This study explores the impact of ownership effects triggered by teachers' involvement in different ownership acquisition styles on the learning performance of Chinese children. Hypothesis 4 states that regardless of ownership acquisition style based on choice or assigning, the self-ownership condition significantly outperforms the teacher-ownership condition, and the teacher-ownership condition significantly outperforms the other-ownership condition. The exploration of these issues not only helps deeply understand how Chinese children build their relationships with themselves and others but also provides new ideas from the perspective of ownership effects to explore the role of interdependent culture in enhancing children's learning performance. This has important theoretical and practical significance for promoting traditional Chinese culture and educational reforms in the Chinese style.

## 2 Experiment 1: How mother-ownership influences children's learning performance in different ownership acquisition styles

### 2.1 Method

#### 2.1.1 Experimental design

A two-factor mixed experimental design was adopted: 2 (ownership acquisition style: choosing and assigning) × 3 (ownership condition: self, mother, and others). In this, the ownership acquisition style was the intergroup variable and ownership condition was the intragroup variable. The dependent variable was the participants' correct free recall rate (sum of correct free recall of drawings and labels).

#### 2.1.2 Participants

Students from five primary schools in three grade (mean = 8.37 years old) were invited to participate in the study, and 77 children provided written parental consent. Therefore, the experiment included forty participants (18 girls) in the group with the chosen ownership acquisition style and thirty seven participants (15 girls) in the group with the assigned ownership acquisition style. Before conducting all three experiments, caregivers' consent was obtained. This study was approved by the Ethics Committee of Hubei Engineering University (IRB NO. H2025001). If the participants experienced discomfort during the experiment, they were allowed to withdraw.

#### 2.1.3 Materials

We used nine cards consisting of drawings and false word labels from the work of Wu et al. (2019). 9 drawings were from the research of Cunningham et al. (2018), which were selected according to that they were drawn in a single line and did not resemble any object in daily life. The nine false-word labels were composed of two common Chinese characters. The Chinese characters were adopted from the Corpus Word POS list, which is a frequency table for word types in modern Chinese corpora. They chose the 100 most frequent Chinese characters (all with a frequency greater than 100/million) as morphemes to form new words. Thirty third-grade primary school students who did not participate in the formal experiment were asked to pronounce and form words using the selected characters. Finally, eighty characters with correct pronunciation and word formation were selected as target words, with an average frequency of 513/million and an average number of strokes of 4.7. The target words were then paired, and another 15 third-grade primary school students rated the degree of the fake word “fake”. Finally, nine false-word labels were selected as experimental materials.

Each card was rated before the experiment, with a drawing on top and a false word label (“水月,” “在个,” “以美”) below. All cards were divided into nine groups. Each group included three cards, and the three ownership conditions were fully balanced in the learning and recognition stages.

### 2.2 Procedure

#### 2.2.1 Chosen ownership acquisition styles

The children were tested individually by a single experimenter. The child sat in front of a table in a quiet area at the school. Three large baskets were positioned in front of the child. Nine small square cards were presented face-up, each showing an outline of a novel shape associated with a false word label (“水月,” “在个,” and “以美”). The children were told that they would play a game with nine unusual shapes. Three were owned by the child, three by a stranger, and three by their mothers. The participants were given three baskets to choose cards, and the order of who chose the first or last card was fully balanced during the experiment. When the participants selected a shape card, they placed it face up in “their” basket (in front of them). Once each basket had three cards, the experimenter read aloud the names of the shapes on her cards, with the child and mother (order counterbalanced across participants), reiterating the ownership (e.g., “So, your cards are “水月,” “在个,” and “以美” [pointing]…” The experimenter noted the location of all nine cards on a record sheet, placed the cards into an envelope, and removed it from sight.

The experimenter created a brief delay while storing the stimuli. The participants were then given a blank sheet of paper and asked to draw as many novel shapes and write as many novel labels as they could remember. The children were allowed as much time as they wanted to complete the task. When they had not produced any drawings or writing for a 20-sec period, the experimenter asked if they were finished. If they said no, the waiting period was repeated. If they said yes, they were thanked, debriefed by the experimenter, and returned to the classroom.

#### 2.2.2 Assigned ownership acquisition styles

Each child was tested individually by an experimenter at a table with three baskets and nine labeled cards. The children were told that they would play a game in which they had to sort nine novel shapes into three colored baskets positioned in front of them. Three shapes were owned by the child, three by strangers, and three by their mother. The experimenter explained that ownership would be assigned based on color matching: a card with a red border would be assigned to the person who owned the red basket, a card with a yellow border would be assigned to the person who owned the yellow basket, and a card with a green border would be assigned to the person who owned the green basket. The assignment of shapes to the ownership condition and of color to the owner was counterbalanced across participants, and the ownership of baskets was explained to the children when they were introduced to the sorting task. After the instructions were provided, the procedures across the ownership and control conditions were identical. The children were asked to place the cards face-up in the correct basket. The experimenter then verbalized the labels of the three categories of shapes (i.e., self-owned, stranger other-owned, and mother-owned) in an order counterbalanced across participants. The cards were then placed in an envelope and removed from sight. As with the chosen ownership condition, the children were given a blank sheet of paper and asked to draw any shape and write labels that they could remember. They were allowed as long as they wanted to complete the task. When the child had not produced any drawing or writing for a 20-sec period, the experimenter asked if they had finished. If the child said no, the waiting period was repeated. If the child answered yes, they were thanked, debriefed by the experimenter, and returned to the classroom.

### 2.3 Statistical analysis

We conducted a 2 (ownership acquisition style: choosing and assigning) × 3 (ownership condition: self, mother, and others) repeated-measures ANOVA to determine the correct free recall rate. We used the Bonferroni correction to address multiple comparisons with adjusted p-values for *post hoc* tests to compare two or three specific groups. Statistical significance was set at *p* < 0.05. SPSS 20.0 was used for all statistical analyses.

### 2.4 Results

All children's drawings were scored by two independent judges who were blinded to the nature of the experiment and shape ownership conditions. This study referred to the scoring criteria from the previous study (Cunningham et al., 2018; Wu et al., 2019). For example, the child was awarded one point for every drawing that could be identified as one of the original nine shapes and one point for each correctly remembered label (regardless of whether the corresponding shape was presented). Thus, the potential recall ranged from 0 to 18 shape stimuli and was split across the three ownership conditions. The score for each ownership condition was the average score given by all judges for this ownership condition. Cohen's κ showed a substantial level of agreement between the two judges, significantly above chance (κ =.720 (95% CI 0.56 to 0.88), *p* < 0.001). The children also produced unscored attempts, which were often close to the original picture or label but not sufficiently matching to be scored as correct by the judges. These errors comprised 18.0% of total productions.

**Figure 1:**
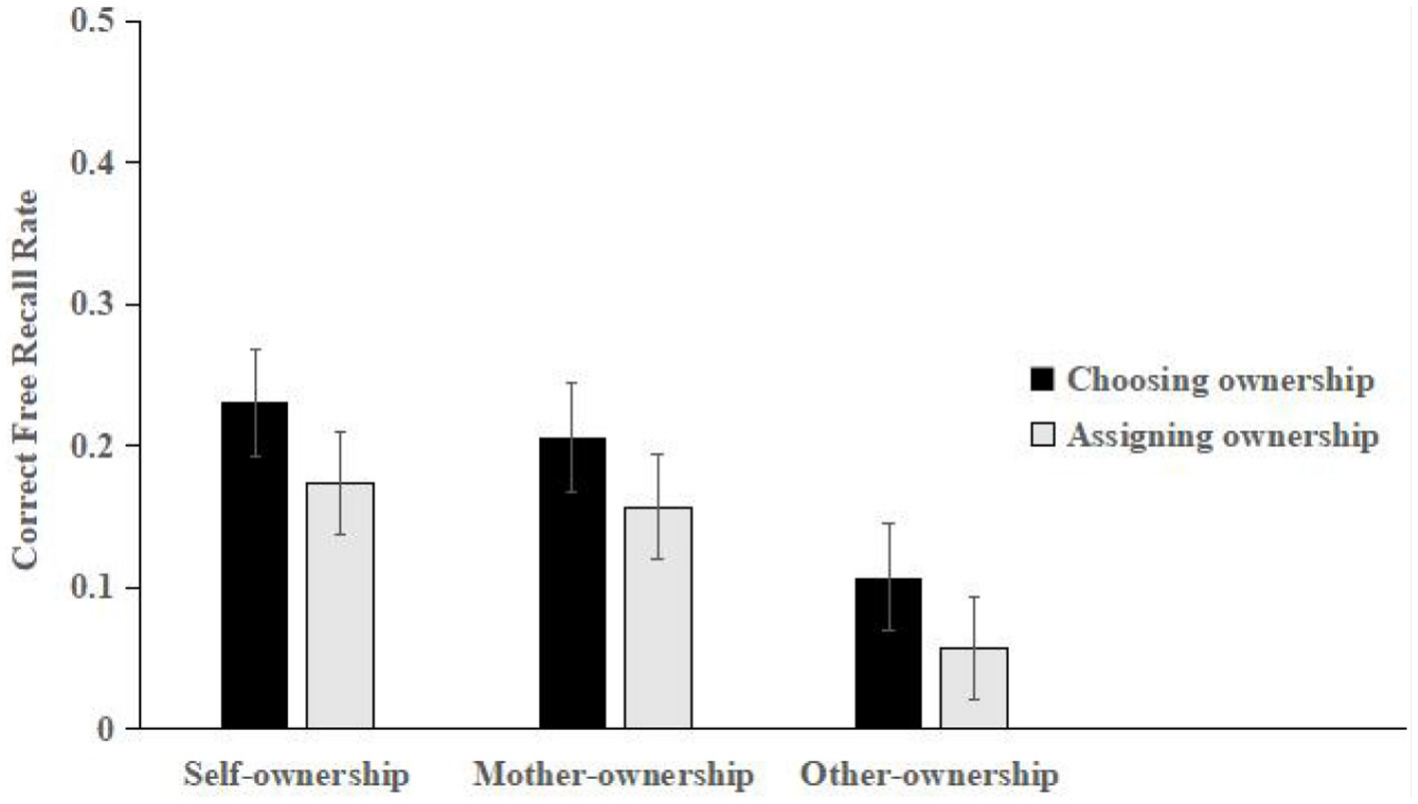
Mean correct free recall rates for the self-, mother-, and others-ownership stimuli in Experiment 1 (error bars represent one standard error of the mean).

Proportionate recall scores were calculated by dividing the number of self-, others-, and mother-owned stimuli correctly recalled by the number of items presented in each condition. The mean scores for each of the three conditions are shown in Figure 1.

The mean scores were subjected to a mixed 2 (ownership acquisition style: choosing and assigning) × 3 (ownership condition: self, mother, and others) design. A repeated measures analysis of variance (ANOVA) confirmed that the main effect of ownership acquisition (*F*_(1, 74)_ = 4.743, *p* < 0.05, $\eta_{p}^{2}$ = 0.097) was significant, and the free recall of chosen ownership acquisition styles was more than that of assigned ownership acquisition styles. The main effect of the ownership condition (*F*_(2, 148)_ = 2.993, *p* = 0.055, $\eta_{p}^{2}$ = 0.064) was significant. A *post-hoc* analysis revealed that significantly more free recall was correctly reproduced for the self- (*p* < 0.001) and mother-owned (*p* < 0.05) stimuli than for the others-owned stimuli, which did not differ significantly from each other (*p* > 0.05). However, there was no significant interaction between the ownership acquisition style and ownership condition (*F*_(2, 148)_ = 0.008, *p* > 0.05).

Experiment 1 found no significant difference in the free recall performance between the self- and mother-ownership conditions, but both were higher than the others-ownership condition, showing obvious self- and mother-ownership effects in the Chinese children's memory. The experimental results also showed that the free recall performance in the mother-ownership condition showed the same memory advantage as that in the self-ownership condition, which supported hypothesis 2 and was consistent with the results of previous studies (Sparks et al., 2016). This indicated that the involvement of the mother and self improved Chinese children's learning performance. The study also found that the free recall performance for the chosen ownership acquisition style condition was significantly higher than that for the assigned ownership acquisition condition, which has verified hypothesis 1. The difference between the two styles of ownership acquisition was that the choice-based ownership method had an additional process of choosing cards independently, unlike the assigned ownership method; this finding supports the occurrence mechanism of ownership based on self-choice (Cunningham et al., 2011, 2008).

## 3 Experiment 2: How friend-ownership influences children's learning performance in different ownership acquisition styles

### 3.1 Method

#### 3.1.1 Experimental design

We replaced the mother-ownership condition in ***Experiment 1*** with the friend-ownership condition and kept all other aspects the same as in ***Experiment 1***.

#### 3.1.2 Participants

Students from five primary schools in three grade (mean = 8.42 years old) were invited to participate in the study, and 80 children provided written parental consent. Therefore, the experiment included forty participants (20 girls) in the chosen ownership group and 40 participants (19 girls) in the assigned ownership group.

#### 3.1.3 Materials

The materials used were identical to those used in Experiment 1.

### 3.2 Procedure

The procedure was mostly identical to that of Experiment 1, apart from replacing the mother-ownership condition with the friend-ownership condition.

### 3.3 Statistical analysis

The methods of statistical analysis used were identical to those used in ***Experiment 1***.

### 3.4 Results

**Figure 2:**
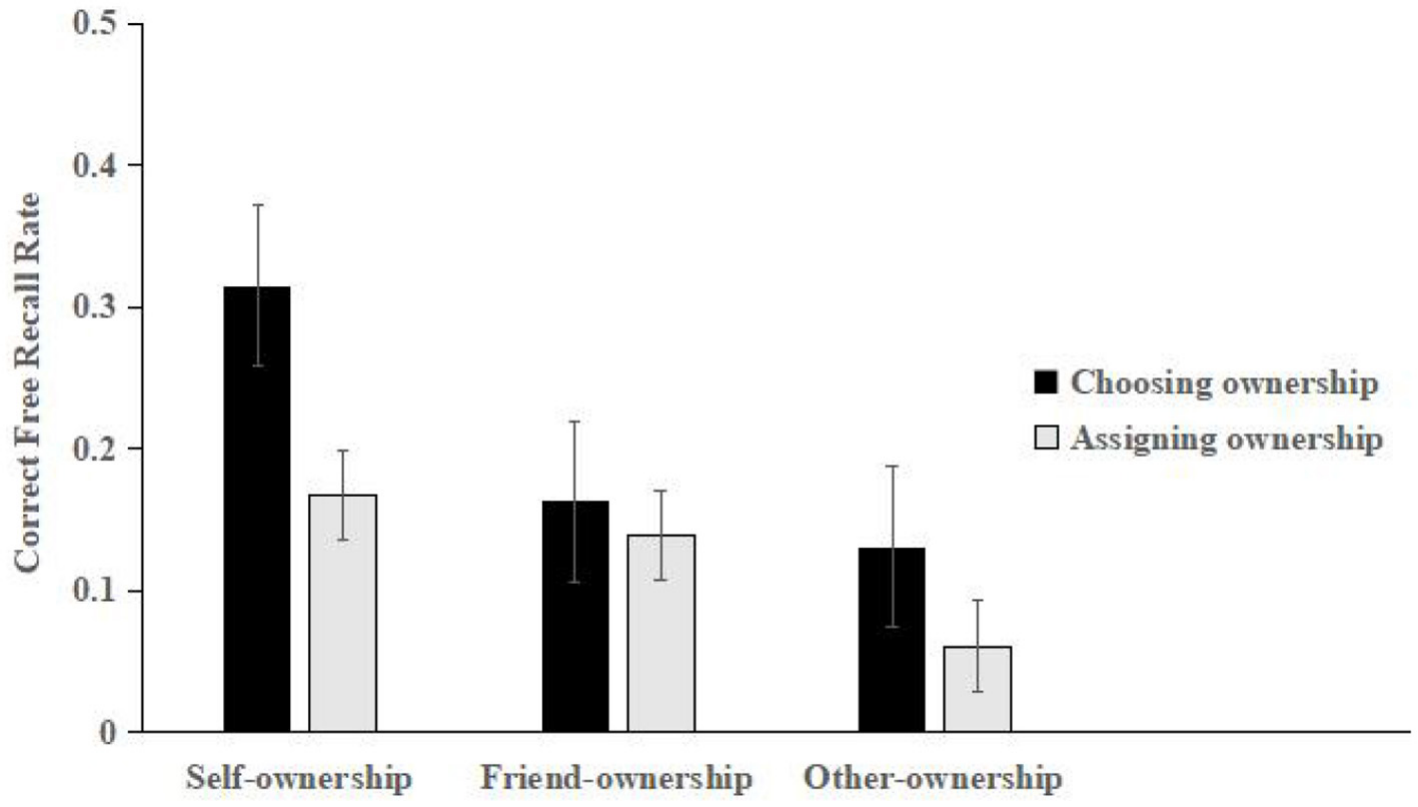
Mean correct free recall rates for the self-, friend-, and others-ownership stimuli in Experiment 2 (error bars represent one standard error of the mean).

The scoring and rating criteria for the correct recall rate in the self-, friend-, and other-ownership conditions were the same as those in ***Experiment 1***. The mean scores for each of the three conditions are shown in Figure 2.

The mean scores were subjected to a mixed 2 (ownership acquisition style: choosing and assigning) × 3 (ownership condition: self, friend, and others) design. A repeated measures ANOVA confirmed that the main effect of ownership acquisition (*F*_(1, 78)_ = 4.743, *p* < 0.001, $\eta_{p}^{2}$ = 0.186) was significant, and the free recall of the chosen ownership acquisition styles was more than that of the assigned ownership acquisition styles. The main effect of ownership condition (*F*_(2, 156)_ = 12.968, *p* < 0.001, $\eta_{p}^{2}$ = 0.188) was significant. A *post-hoc* analysis revealed that significantly more free recall was correctly reproduced for the self- (*p* < 0.001) and friend-owned (*p* < 0.01) stimuli than for the others-owned stimuli, whereas significantly more free recall was correctly reproduced for the self-owned stimuli than for the friend-owned stimuli (*p* < 0.05). However, there was no significant interaction between ownership acquisition style and ownership condition (*F*_(2, 156)_ = 2.292, *p* > 0.05).

Experiment 2 found that the free recall performance in the self- and friend-ownership condition was significantly higher than in the others-ownership condition, and free recall performance in the self-ownership condition was also significantly higher than in the friend-ownership condition, which supported hypothesis 3. These findings highlighted obvious self- and friend-ownership effects. In addition, the learning performance of the self-ownership effect was better than that of the friend-ownership effect. This indicated that the learning effect of peer relationships in the participants' learning process was not as crucial as that of self-involvement but was conducive to improving Chinese children's learning performance, which is consistent with previous studies in the field of memory (Wu and Zhou, 2013; Yuan, 2007). The free recall performance of ownership acquired through choosing was significantly higher than that acquired through assignments, which has verified hypothesis 1 and also supported the idea that the ownership effect is a self-choice-based mechanism (Cunningham et al., 2011, 2008).

## 4 Experiment 3: How teacher-ownership effect influences children's learning performance in different ownership acquisition styles

### 4.1 Method

#### 4.1.1 Experimental design

We replaced the mother-ownership condition in ***Experiment 1*** with the teacher-ownership condition and kept all other aspects the same as in ***Experiment 1***.

#### 4.1.2 Participants

Students from five primary schools in three grade (mean = 8.45 years old) were invited to participate in the study, and eighty children provided written parental consent. Therefore, the experiment included forty participants (20 girls) in the chosen ownership acquisition styles group and forty participants (20 girls) in the assigned ownership acquisition styles group.

#### 4.1.3 Materials

The materials used were identical to those used in Experiment 1.

### 4.2 Procedure

The procedure was mostly identical to that of Experiment 1, apart from replacing the mother-ownership condition with the teacher-ownership condition.

### 4.3 Statistical analysis

The methods of statistical analysis used were identical to those used in ***Experiment 1***.

### 4.4 Results

**Figure 3:**
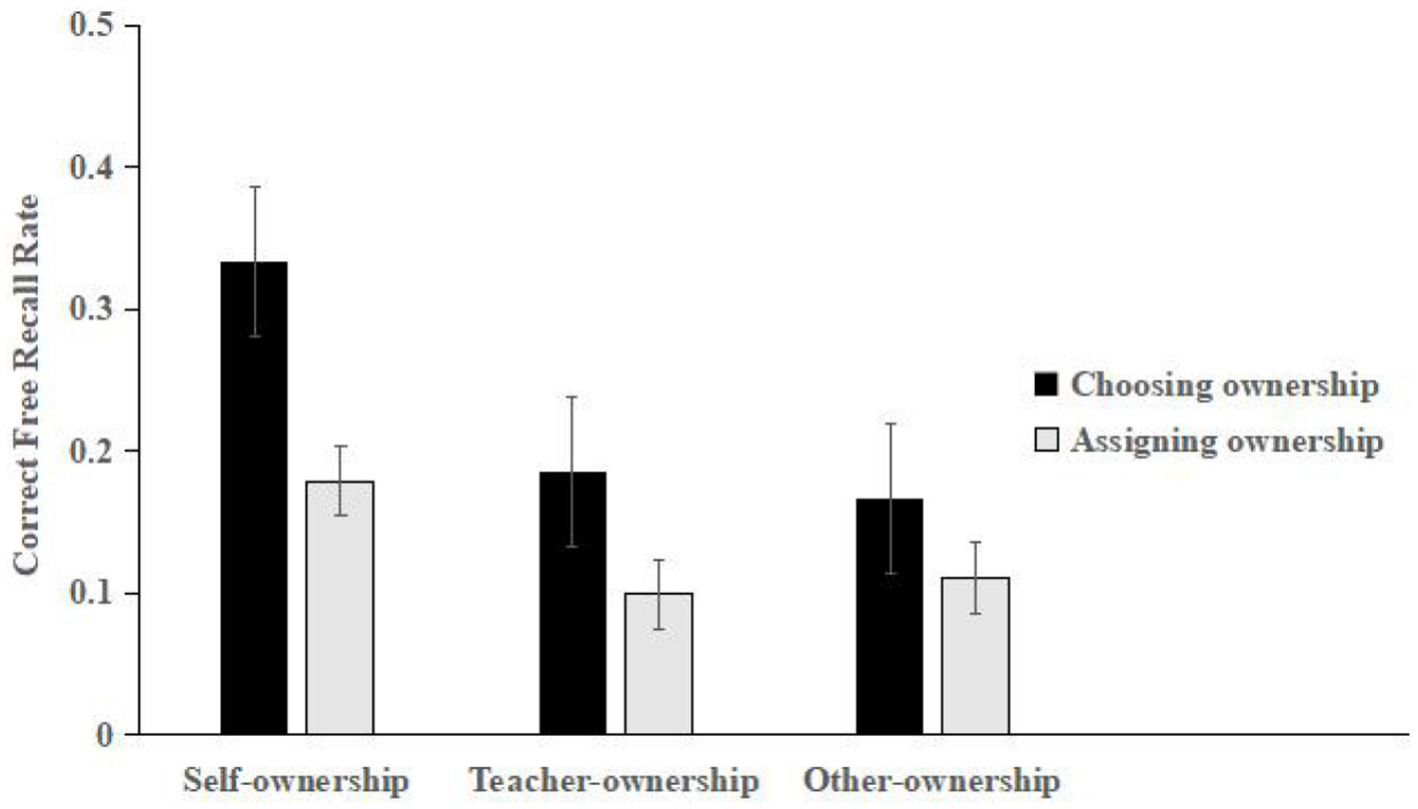
Mean correct free recall rates for the self-, teacher-, and others-ownership stimuli in Experiment 3 (error bars represent one standard error of the mean).

The scoring and rating criteria for the correct recall rate in the self-, teacher-, and other-ownership conditions were the same as those in ***Experiment 1***. The mean scores for each of the three conditions are shown in Figure 3.

The mean scores were submitted to a mixed 2 (ownership acquisition style: choosing and assigning) × 3 (ownership condition: self, teacher, and others) design. A repeated measures ANOVA confirmed that the main effect of ownership acquisition (*F*_(1, 78)_ = 16.065, *p* < 0.001, $\eta_{p}^{2}$ = 0.233) was significant, and the free recall of the chosen ownership acquisition styles was more than that of the assigned ownership acquisition styles. The main effect of the ownership condition (*F*_(2, 156)_ = 7.373, *p* < 0.05, $\eta_{p}^{2}$ = 0.122) was significant. A *post-hoc* analysis revealed that significantly more free recall was correctly reproduced for the self-owned stimuli than the teacher—(*p* < 0.05) and others-owned (*p* < 0.05) stimuli, but there was no significant difference between the others- and friend-owned stimuli (*p* > 0.05). There was no significant interaction between the ownership acquisition style and ownership condition (*F*_(2, 156)_ = 1.054, *p* > 0.05).

In Experiment 3, the results showed that learning performance in the self-ownership condition was significantly higher than in the teacher- and others-ownership conditions. Therefore, the self-ownership effect appeared in memory, indicating that involving the self in learning ownership was conducive to improving the participants' learning performance. However, there was no significant difference between the learning performance in the teacher— and others—ownership conditions, indicating that teacher involvement did not improve the participants' learning performance, which supported hypothesis 4. According to the self-expansion model, people spontaneously seek and maintain close relationships with others, incorporating other individuals with whom they have intimate relationships into their own self-system to achieve enrichment and expansion (Aron et al., 2004). Thus, the self includes relatively important others, such as mothers and friends. Although teachers are important in children's lives, they are not important enough to be part of self-expansion. In addition, ownership acquired by choice was more conducive to improving the participants' learning performance than assigned ownership, which has verified hypothesis 1 and supported the occurrence mechanism of the ownership effect based on self-choice (Cunningham et al., 2011, 2008).

## 5 Discussion

This study compared the effects of ownership acquired styles by assigning and choosing on Chinese children's learning. We found that the learning performance of the chosen ownership acquisition styles in the mother, friend, and teacher-involved conditions was better than that of the assigned ownership acquisition styles. We combined the operation process of the experiment with several studies and presented the following findings.

First, the SDT (Ryan and Deci, 2000a) posits that individuals are positive organisms with positive self-integration. Self-improvement, continuous self-learning behavioral tendencies, and social environmental factors can promote or block the formation and development of positive individual behaviors and healthy psychology. This theory clearly states that basic psychological needs are core concepts of individuals' healthy growth and development. The SDT proposes three basic psychological needs: autonomy, competence, and relatedness. As the chosen ownership style gave children more opportunities for autonomous choice, that is, the participants chose cards according to their own wishes and interests and put them into the basket they chose, they felt less controlled than when the ownership was assigned. Students' internal motivation and learning initiatives were stimulated (Ryan and Deci, 2000b), thereby promoting the improvement of academic performance. Therefore, choice-based ownership may support autonomy more than assigned ownership, which is the root cause of the difference in learning performance between the two ownership styles.

Second, Cunningham et al. (2011) used three experiments to explore the mechanism of ownership and found that only items that individuals selected for themselves had a memory ownership effect. There was no significant difference in memory performance between the items they selected for others or those selected by others for them. This study showed that the memory effect of choice-based ownership was better than the learning effect of assigned ownership. Moreover, a special choice was not enough to create an ownership effect, and this might occur because the individual chose during the process. Therefore, these items were more likely to be perceived as self-related. Beyond this, the participants may be more stimulated to take the initiative when choosing for themselves, enabling their memory to encode more self-related items (Cunningham et al., 2011). In this study, the participants were free to choose the cards according to their own preferences, without excluding the promoting effect of self-knowledge related to autobiographical memory on learning performance. Thus, the learning performance of choice-based ownership was better than that of assignment-based ownership.

Finally, the view of embodied cognition holds that physical activity implicitly affects the cognitive process, which is called the embodiment of physical activity (Davis and Markman, 2012). Some studies have found that, without any ownership guidance, the relationship between ownership and items can be enhanced by simply touching the items. This process may bring a greater sense of control over the items, thus increasing the perceived ownership (Peck and Shu, 2009). In the chosen and assigned ownership style conditions, the participants chose which baskets they would place the cards in. A choice-based ownership style required the individual to choose the basket and cards, whereas the assigned style required the baskets and cards to be assigned based on the corresponding color. The former group exhibited a greater degree of self-participation than the latter. The connection created between the self and ownership was stronger, and attention had a regulatory effect on the establishment of a psychological connection between the two. Attention was selectively, rather than uniformly, distributed, allowing more resources to be allocated to the object to be executed (Brockmole et al., 2013). The process of choosing can activate more attentional resources, thus improving the children's learning performance.

From this, it can be seen that in the process of teaching children, giving full play to students' autonomy in choice is more conducive to improving their learning performance. However, in actual classroom teaching, overly emphasizing students' autonomy in choice may have several drawbacks. First, children may choose items that are attractive or meaningful for themselves, and these items may already have processing biases in memory, such as familiarity or positive emotions (Ashby and Isen, 1999; Ledoux, 1994). If children are attracted to the characteristics of the learning materials themselves, it is difficult to guarantee that the improvement in their learning performance was caused by their choice. Second, although some studies have proven that choice has a positive impact on children's learning performance (Reeve, 2013), others have also indicated that in educational tasks in which children's sensory abilities are insufficient, providing choice does not always benefit the improvement of children's academic performance. They may think that they are the source of their failure (Katz and Assor, 2007). Finally, in the actual teaching process, students' autonomous choices cannot be widely applied to various teaching scenarios because teachers usually need to determine the content to be learned rather than allowing students to drive this process. Giving students the opportunity to have ownership of the choice (allocating the power to assign learning tasks) can make the content that they learn more conducive to promoting the process of children's learning within their zone of proximal development rather than being constrained by children's independent choices. Therefore, the ownership acquisition style based on assigning is more applicable and has better ecological validity in real teaching activities. Future research is necessary to explore whether “who assigns” will have an impact on children's learning performance in different types of intimate-other ownerships in the teaching context based on assigning ownership acquisition style, and particularly for Chinese children, who grow up in an interdependent context, whether the assigning of learning tasks by intimate others (mothers, peers, and teachers) will have an impact on learning performance in different types of intimate-other ownerships.

This study also found that different relationships involving others in children's learning processes had different effects on learning performance. In the condition of parent—child relationship involvement, the mother-ownership condition was conducive to improving children's learning performance, and its effect was comparable to that of self-ownership. After peer relationship involvement, the friend-ownership condition was beneficial for improving children's learning performance but not as beneficial as the self-ownership condition. After the relationship between teachers and students was established, the self-ownership condition could improve children's learning performance, but the teacher condition had no effect on children's learning performance. According to Markus and Kitayama (1991), the self-concept of an individual from an Eastern cultural context is characterized by interdependence, which emphasizes maintaining the interdependent relationship between the self and others, and the self-schema includes relatively intimate individuals such as mothers. However, in Western context, an individual's self-schema is characterized by independence and emphasizes the maintenance of self-integrity and exclusivity, and the Western self does not include intimate others such as mothers. Sparks et al. (2016) explored the reference effect of mothers in different cultural contexts from the perspective of the ownership paradigm. They created a supermarket shopping scene in the experiment and found that individuals from the Western context were significantly more likely to remember items that belonged to themselves than items belonging to mothers and strange others, and a typical ownership effect appeared. For Asian participants, when the items assigned to the self were compared with those assigned to mothers, the results of the items assigned to mothers were better than those assigned to themselves. This indicated that Asian individuals were trying to show the advantage of mother processing in the ownership paradigm. When the items assigned to the self were compared with those assigned to strange-others, performance for the former was better than for the latter, and the ownership effect occurred. From the perspective of ownership, this confirmed the relational self of the Chinese people.

Fei (2008) proposed the “differential pattern” in *Rural China*, which describes the interpersonal patterns of closeness and distance. The interpersonal pattern extends from oneself much like the ripples on the water surface—one circle after another—and divides affinity and distance according to the distance from oneself, that is, the self-construction of the relationship. It is a special form of an interdependent self-construction (Cross et al., 2000) in which individuals construct relationships with others according to proximity. Xu (2005) stated that blood relationships with others, such as parents, are permanent aspects of the Chinese self-concept, indicating that blood relationships have the potential to transcend the limits of close relationships for Chinese people. Communicative others are individuals who seek intimacy and are involved in the process of self-expansion, including teachers, mentors, friends, bosses, and others. These people are important others for the individual but do not include parents or have a preconceived relationship. The higher the actual intimacy between the individual and communicative others, the higher the instrumental value of the other person to the individual, implying that the other person is more important to the individual. This explains the differences in the participants' learning effects between the parent—child and peer relationship conditions. A traditional Chinese teaching is “One day as a teacher, one life as a father,” indicating that the teacher—student relationship is deeply rooted in the self of Chinese children. Yuan (2007) adopted a self-referential research paradigm and used recognition rate as the dependent variable to investigate whether a significant difference existed in memory performance between the self and teachers' reference conditions among Chinese children. However, the recognition results in the teacher-reference condition were significantly higher than those in the other-reference condition, indicating that Chinese children distinguished between themselves and their teachers in their self-construction. Studies have shown that the ownership paradigm is suitable for exploring children's self-processing advantages (Cunningham et al., 2013; Ross et al., 2011). However, despite the adoption of the ownership paradigm, there was no learning performance advantage in the teacher-ownership condition in this study. We proposed that because 8–9-year-old children are young and teachers are communicative others, the children are not yet aware of teachers' instrumental value and importance; therefore, these aspects are not yet incorporated into the deeper self-structure.

The results of this study showed that, for the participants, their mothers' involvement in their learning performance was as beneficial as self-involvement. Although learning performance in the friend-involvement condition was not as high as in the self-involvement condition, an improvement was still observed. However, the worst learning performance was obtained in the teacher-involvement condition, which indicates the high value of applying the ownership education model to the teaching process of Chinese children with interdependent self-characteristics.

## 6 Limitations

This study had several limitations that should be addressed in future research. First, the relatively small sample size with a narrow age range resulted in relatively small effect sizes, which increased the risk of Type I errors and limited the generalizability of the findings. This study involved only 77–80 children per experiment within the age range of 7–9 years, making it difficult to generalize the findings to other age groups or broader contexts. Additionally, the cross-sectional design did not allow us to observe the developmental effects of ownership over time. In future research, it will be necessary to conduct replication studies with larger and more diverse samples and longitudinal studies to understand how the ownership effect influences the learning performance of individuals at different age stages. Second, this study was conducted only with Chinese children in an interdependent cultural context. There is a lack of comparative research with children from independent cultural contexts, and the research conclusions obtained lack strong ecological validity. Cross-cultural comparisons with children from individualistic cultures enrich our understanding of how cultural contexts influence learning performance. Third, previous studies have shown that in interdependent cultural contexts, the closeness of relationships with others can affect memory performance related to the self (Xia et al., 2015; Zhou and Su, 2008). Future research needs to further explore the impact of the degree of closeness with relational others (such as mothers, teachers, and friends) on the ownership effect as well as examine variables such as students‘ intrinsic motivation and engagement levels so that the research results can better guide students' learning practices.

## 7 Conclusion

This study has implications for basic education. First, this research will help educators in interdependent cultural contexts realize that ownership teaching is a teaching strategy that needs to be widely disseminated, especially ownership teaching methods based on choice. The simplicity, efficiency and economy of its operation process will inspire educational researchers to fully explore the potentially more valuable tools that may exist in this “educational toolbox.” Second, relying solely on school education is insufficient to enhance the learning performance of Chinese children influenced by an interdependent culture. Therefore, it is necessary to leverage the roles of family education and peer-assisted learning. This also indicates that in the process of educational reform and policy formulation for Chinese children, the influence of culture should be fully considered.

## Data Availability

The raw data supporting the conclusions of this article will be made available by the authors, without undue reservation.

## References

[B1] Aron A. McLaughlin-Volpe T. Mashek D. Lewandowski G. Wright S. C. Aron E. N. (2004). Including others in the self. Eur. Rev. Soc. Psychol. 15, 101–132. 10.1080/10463280440000008

[B2] Ashby F. G. Isen A. M. (1999). A neuropsychological theory of positive affect and its influence on cognition. Psychol. Rev. 106:529. 10.1037/0033-295X.106.3.529 10467897

[B3] Barlas Z. Hockley W. E. Obhi S. S. (2018). Effects of free choice and outcome valence on the sense of agency: evidence from measures of intentional binding and feelings of control. Exp. Brain Res. 236, 129–139. 10.1007/s00221-017-5112-3 29080100

[B4] Beggan J. K. (1992). On the social nature of nonsocial perception: the mere ownership effect. J. Personal. Soc. Psychol. 62, 229–237. 10.1037/0022-3514.62.2.229

[B5] Brockmole J. R. Davoli C. C. Abrams R. A. Witt J. K. (2013). The world within reach: effects of hand posture and tool use on visual cognition. Curr. Dir.Psychol. Sci. 22, 38–44. 10.1177/0963721412465065

[B6] Cloutier J. Neil Macrae C. (2008). The feeling of choosing: self-involvement and the cognitive status of things past. Conscious. Cogn. 17, 125-135. 10.1016/j.concog.2007.05.010 17611123

[B7] Cross S. E. Bacon P. L. Morris M. L. (2000). The relational-interdependent self-construal and relationships. J. Personal. Soc. Psychol. 78:791. 10.1037/0022-3514.78.4.791 10794381

[B8] Cunningham S. J. Brady-Van den Bos M. Turk D. J. (2011). Exploring the effects of ownership and choice on self-memory biases. Memory 19:449. 10.1080/09658211.2011.584388 21864211

[B9] Cunningham S. J. Scott L. Hutchison J. Ross J. Martin D. (2018). Applying self-processing biases in education: Improving learning through ownership. J. Appl. Res. Mem. Cogn. 7, 342–351. 10.1016/j.jarmac.2018.04.004

[B10] Cunningham S. J. Turk D. J. Macdonald L. M. Neil Macrae C. (2008). Yours or mine? Ownership and memory. Conscious. Cogn. 17, 312–318. 10.1016/j.concog.2007.04.003 17543540

[B11] Cunningham S. J. Vergunst F. Macrae C. N. Turk D. J. (2013). Exploring early self-referential memory effects through ownership. Br. J. Dev. Psychol. 31, 289–301. 10.1111/bjdp.12005 23901843

[B12] Davis J. I. Markman A. B. (2012). Embodied cognition as a practical paradigm: introduction to the topic, the future of embodied cognition. Top. Cogn. Sci. 4, 685–691. 10.1111/j.1756-8765.2012.01227.x 23060128

[B13] Deci E. L. Ryan R. M. (1980). The empirical exploration of intrinsic motivational processes. Adv. Exp. Soc. Psychol. 13, 39–80.

[B14] Deci E. L. Ryan R. M. (1985). The general causality orientations scale: self-determination in personality. J. Res. Pers. 19, 109–134.

[B15] Dellarosa D. Bourne L. E. (1984). Decisions and memory: differential retrievability of consistent and contradictory evidence. J. Verbal Learn. Verbal Behav. 23, 669–682. 10.1016/S0022-5371(84)90410-9

[B16] Fei X. T. (2008). Rural China. Beijing: People Press. 25–34.

[B17] Feng T. Zhao W. Donnay G. F. (2013). The endowment effect can extend from self to mother: evidence from an fMRI study. Behav. Brain Res. 248, 74–79. 10.1016/j.bbr.2013.04.005 23588273

[B18] Gaschler R. Nattkemper D. (2012). Instructed task demands and utilization of action effect anticipation. Front. Psychol. 3:578. 10.3389/fpsyg.2012.00578 23431066 PMC3577050

[B19] Guan Y. Chi Y. (2006). The effects of self-reference and friend-reference on the memory of personality traits. Psychol. Sci. 29:3. 10.16719/j.cnki1671-6981.2006.02.052

[B20] Haggard P. (2003). “Conscious awareness of intention and of action,” in Agency and self-Awareness, 111–127.

[B21] Halpin J. A. Puff C. R. Mason H. F. Marston S. P. (1984). Self-reference encoding and incidental recall by children. Bull. Psychon. Soc. 22, 87–89. 10.3758/BF033337706747551

[B22] Herwig A. Horstmann G. (2011). Action-effect associations revealed by eye movements. Psychon. Bull. Rev. 18, 531–537. 10.3758/s13423-011-0063-3 21327975

[B23] Hommel B. Lippelt D. P. Gurbuz E. Pfister R. (2017). Contributions of expected sensory and affective action effects to action selection and performance: evidence from forced- and free-choice tasks. Psychon. Bull. Rev. 24, 821–827. 10.3758/s13423-016-1139-x 27519674 PMC5486880

[B24] Kahneman D. (2003). Maps of bounded rationality: psychology for behavioral economics. Am. Econ. Rev. 93, 1449–1475. 10.1257/00028280332265539227928763

[B25] Katz I. Assor A. (2007). When choice motivates and when it does not. Educ. Psychol. Rev. 19, 429–442. 10.1007/s10648-006-9027-y

[B26] Kitayama S. Uskul A. K. (2011). Culture, mind, and the brain: current evidence and future directions. Ann. Rev. Psychol. 62, 419–449. 10.1146/annurev-psych-120709-145357 21126182

[B27] Knoblich G. Prinz W. (2001). Recognition of self-generated actions from kinematic displays of drawing. J. Exp. Psychol. Hum. Percept. Perform. 27, 456-465. 10.1037/0096-1523.27.2.456 11318059

[B28] Ledoux J. E. (1994). Emotion, memory and the brain. Sci. Am. 270, 50–57. 10.1038/scientificamerican0694-50 8023118

[B29] Li J. Z. Hu Y. M. (2021). Study on the relationship between parents' learning participation and students' academic achievement: A mediated regulatory model based on parent–child relationship and learning self-confidence. J. East China Norm. Univ. 39, 72–83. 10.16382/j.cnki.1000-5560.2021.07.007

[B30] Liu Z. Y. Guo R. (2020). The impact of migrant children's peers on students' academic achievement: Based on analysis of three districts in Beijing. J. Educ. Econ. 1, 64-76.

[B31] Marcel A. J. (2003). “The sense of agency: awareness and ownership of action,” in Agency and Self-Awareness: Issues in Philosophy and Psychology, eds. Roessler J. Eilan N. (Oxford: Clarendon Press).

[B32] Markus H. R. Kitayama S. (1991). Cultural variation in the Self-Concept. New York, NY: Springer. 10.1007/978-1-4684-8264-5_2

[B33] Mead G. H. (1999). Mind, self and society. J. High. Educ. 70, 620–620. 10.2307/2649240

[B34] Peck J. Shu S. B. (2009). The effect of mere touch on perceived ownership. J. Consum. Res. 36, 434–447. 10.1086/598614

[B35] Pullyblank J. Bisanz J. Scott C. Champion M. A. (1985). Developmental invariance in the effects of functional self-knowledge on memory. Child Dev. 56, 1447–1454. 10.2307/113046417087552

[B36] Reeve J. (2013). How students create motivationally supportive learning environments for themselves: the concept of agentic engagement. J. Educ. Psychol. 105:579. 10.1037/a0032690

[B37] Repp B. H. Knoblich G. (2004). Perceiving action identity: How pianists recognize their own performances. Psychol. Sci. 15, 604–609. 10.1111/j.0956-7976.2004.00727.x 15327631

[B38] Rescher N. (1960). The problem of a logical theory of belief statements. Philos. Sci. 27, 88–95. 10.1086/287715

[B39] Ross J. Anderson J. R. Campbell R. N. (2011). I. Why investigate mnemonic self-reference effects in preschoolers? Monogr. Soc. Res. Child Dev. 76, 1–16. 10.1111/j.1540-5834.2011.00614.x

[B40] Rotter J. B. (1966). Rotter's Internal-External Control Scale. APA PsycTests.

[B41] Ryan R. M. Deci E. L. (2000a). Intrinsic and extrinsic motivations: classic definitions and new directions. Contemp. Educ. Psychol. 25, 54–67. 10.1006/ceps.1999.1020 10620381

[B42] Ryan R. M. Deci E. L. (2000b). Self-determination theory and the facilitation of intrinsic motivation, social development, and well-being. Am. Psychol. 55:68. 10.1037/0003-066X.55.1.68 11392867

[B43] Sebanz N. Lackner U. (2007). Who's calling the shots? Intentional content and feelings of control. Conscious. Cogn. 16, 859–876. 10.1016/j.concog.2006.08.002 17045811

[B44] Sidarus N. Vuorre M. Haggard P. (2017). How action selection influences the sense of agency: an ERP study. Neuroimage 150, 1–13. 10.1016/j.neuroimage.2017.02.015 28188916

[B45] Sittenthaler S. Traut-Mattausch E. Jonas E. (2015). Observing the restriction of another person: vicarious reactance and the role of self-construal and culture. Front. Psychol. 6:1052. 10.3389/fpsyg.2015.01052 26300795 PMC4523787

[B46] Sparks S. Cunningham S. J. Kritikos A. (2016). Culture modulates implicit ownership-induced self-bias in memory. Cognition 153, 89–98. 10.1016/j.cognition.2016.05.003 27164187

[B47] Sui J. Sun Y. Peng K.. (2014). The automatic and the expected self: separating self- and familiarity biases effects by manipulating stimulus probability. Atten. Percept. Psychophys. 76, 1176–1184. 10.3758/s13414-014-0631-5 24519433

[B48] Taylor S. E. (1989). Positive Illusions: Creative Self-Deception and the Healthy Mind. Basic Books; Hachette Book Group.

[B49] Ullmann-Margalit E. Morgenbesser S. (1977). Picking and choosing. Soc. Res. 44, 757–785.

[B50] Wenzlaff R. M. LePage J. P. (2000). The emotional impact of chosen and imposed thoughts. Personal. Soc. Psychol. Bull. 26, 1502–1514. 10.1177/0146167200261200516396848

[B51] Wu H. Wang Y. Hu X. Zhang H. Chen Y. Guo D.. (2021). The Influence of the Survival Status of the Mother on the Mother Reference Effect Among Chinese People. Chinese

[B52] Wu H. F. Liu H. S. Chen Y. Y. Jiang Y. Z. (2019). The effect of ownership on children's learning performance. Educ. Res. Exp. 3, 78–84.

[B53] Wu H. F. Xiao H. P. Hu X. Y. Huang F. K. Peng C. (2020). The mechanism of ownership effect and its implications for early childhood education. Educ. Res. Exp. 5, 85–90.36217271

[B54] Wu H. F. Zhou A. B. (2013). An empirical study on the friend reference effect of Chinese young college students. Psychol. Behav. Res. 11, 380–386.

[B55] Wuyun G. Wang J. Zhang L. Wang K. Yi L. Wu Y. (2020). Actions speak louder than words: the role of action in self-referential advantage in children with autism. Aut. Res. 13, 810–820. 10.1002/aur.2274 32011827

[B56] Xia R. Jin R. Zhao H. Niu B. (2015). The moderating effect of actual intimacy on the self-concept's inclusion of different types of others. Chin. J. Clin. Psychol. 23, 843–847.

[B57] Xu K. (2022). The ownership effect in children with autism: The role of action and choice (Doctoral dissertation), Northeast Normal University.

[B58] Xu S. G. (2005). Cross-cultural self-perspective. See Yang Y. Y. Chin. Soc. Psychol. Rev. Beijing: Social Sciences Academic Press 1–20.

[B59] Yuan Z. X. (2007). A Developmental Study on The Self-Reference Effect of Children's Memory. Zhejiang Normal University.

[B60] Zhao J. Wang Y. Kong F. (2014). Exploring the mediation effect of social support and self-esteem on the relationship between humor style and life satisfaction in Chinese college students. Personal. Individ. Diff. 64, 126–130. 10.1016/j.paid.2014.02.026

[B61] Zhou A. B. Liu P. R. Shi Z. Zhang P. Y. Wu H. F. Li Q. (2010). A study of self-referential effects in four-year-old children. Psychol. Dev. Educ. 26, 239–244. 10.16187/j.cnki.issn1001-4918.2010.03.009

[B62] Zhou L. Su Y. (2008). The intimacy on the reference effect of romantic partners. Acta Psychologica Sinica 40, 487–495. 10.3724/SP.J.1041.2008.0048737113526

[B63] Zhu Y. Zhang L. Fan J. Han S. (2007). Neural basis of cultural influence on self-representation. Neuroimage 34, 1310–1316. 10.1016/j.neuroimage.2006.08.047 17134915

